# Characteristics of Oral Mucosal Lesions and Their Association With Socioeconomic Status and Systemic Health: A Cross-Sectional Study of Consecutively Collected Oral Medicine Clinic Data in a Remote Rural Area of China

**DOI:** 10.3389/fpubh.2022.897814

**Published:** 2022-05-23

**Authors:** Hui Yao, Qiongyue Zhang, Qianqian Song, Mingshan Liu, Guoyao Tang

**Affiliations:** ^1^Department of Oral Medicine, Shanghai Ninth People's Hospital, Shanghai Jiao Tong University School of Medicine, Shanghai, China; ^2^College of Stomatology, Shanghai Jiao Tong University, Shanghai, China; ^3^National Center for Stomatology, Shanghai, China; ^4^National Clinical Research Center for Oral Diseases, Shanghai, China; ^5^Shanghai Key Laboratory of Stomatology, Shanghai, China; ^6^Department of Stomatology, The People's Hospital of Xiangyun Affiliated With Dali University, Yunnan, China

**Keywords:** oral mucosa, ulcer, burning mouth syndrome, lichen planus, rural, socioeconomic status, systemic health

## Abstract

**Objectives:**

Epidemiological data of oral mucosal lesions (OMLs) are required to develop practical oral care policies. However, limited data are available for rural areas in China. We aimed to estimate the spectrum and frequency of OMLs and to identify their associated socioeconomic status (SES) and systemic health in a remote rural area in Yunnan, China.

**Methods:**

We screened patients for OMLs in an oral medicine clinic in rural Yunnan, China, from August 2020 to February 2021. OMLs were documented. SES, including the highest education level achieved and the previous month's household income, as well as the patient's systemic health, including a history of smoking, alcohol use, and chronic disease, were obtained from the Medical History/Health Questionnaire Form and patient medical records.

**Results:**

A total of 146 patients were found to have OMLs. The most frequent OML was aphthous ulcer (*n* = 41, 28.1%), followed by burning mouth syndrome (BMS) (*n* = 16, 11.0%), viral ulcer (*n* = 14, 9.6%), and oral lichen planus (OLP) (*n* = 9, 6.2%). In these patients, the most common chronic diseases were sleep apnea (*n* = 35, 24.0%), hypertension (*n* = 23, 15.8%), bronchitis (*n* = 16, 11.0%), reflux (including gastroesophageal reflux disease) (*n* = 12, 8.2%), and arthritis (*n* = 11, 7.5%). On adjusted regression, the patients without chronic diseases had a lower risk of BMS than those with chronic diseases [odds ratio (OR), 0.2; 95% confidence interval (CI), 0.03–0.9]. Age was an independent protective factor for viral ulcers (OR, 1.0; 95% CI, 0.9–1.0). Patients with low-income levels had a lower risk of OLP than those with high-income levels (OR, 0.2; 95% CI, 0.05–0.9).

**Conclusions:**

Our oral medicine clinic data in remote Yunnan, China, showed the most frequent OML was aphthous ulcer, which was followed by BMS, viral ulcer, and OLP. Oral care policies should be prioritized among patients with aphthous ulcer. Preventive strategy of BMS should be targeted to people with chronic disease for health equalities. Therefore, an individualized strategy for interventions of OMLs might be optimal, based on the specific epidemiologic characteristics in rural settings.

## Introduction

Oral mucosal lesions (OMLs) include various disorders involving the oral mucosa, affecting 4.9–64.7% of people in the general population worldwide (1). Some lesions can impair chewing, swallowing, and speaking functions (2), while others may influence systemic health (3) or progress to oral cancer (4). The Chinese government released the Healthy China 2030 blueprint to guide the healthcare promotion (5). Oral health is an important part of this blueprint. To promote oral health, manage oral diseases, and develop an oral care strategy, it is urgent for governments to understand local epidemiological characteristics of OMLs.

In the rural areas, patients with OMLs often suffer more due to their difficulty with accessing appropriate oral healthcare in a timely manner. The epidemiology of OMLs is also not well-characterized in remote rural areas due to a lack of local oral specialists. Recently, the Shanghai and Yunnan governments organized a program for oral specialists to temporarily work in these rural areas and perform free oral examinations in the local population. This provides an opportunity to understand the characteristics of OMLs in local rural areas. Xiangyun County eliminated poverty in 2018 (6), with the per capita disposable income of 37,689 Yuan (4,997 Euro) in 2019 (7), equivalent to 6,000-Yuan per month per household from two full-time incomes. Her population reached 406,642 in 2020 (8).

In this study, we mainly aimed to determine the spectrum and frequency of OMLs from an oral medicine clinic data in a remote rural area in Yunnan, China. We additionally aimed to assess their possible associations with socioeconomic status (SES) and systemic health.

## Methods

### Study Setting and Participants

The present study was performed in a rural Oral Medicine Clinic ran by the Department of Stomatology, the People's Hospital of Xiangyun affiliated with Dali University, China. This clinic included an oral medicine specialist who stationed, practiced, and trained two general dentists there between August 2020 and February 2021. With the support of the poverty-alleviation program collaborated by Shanghai and Yunnan, the specialist worked there and provided free oral examinations. The ethics committee of the People's Hospital of Xiangyun approved this study (no. 2020069) on April 30, 2020. All patients and/or their guardians signed informed consent. All consecutive visitors to our oral medicine clinic with a chief complaint of oral mucosal problems were eligible for the study. But visitors without any abnormal oral mucosal findings were excluded.

### Data Collection

A single-clinic cross-sectional study was performed. The participants enrolled in this study were sent by the receptionists and the general dentists at the department of stomatology. We included all consecutive 146 patients, who arrived for their first oral medicine visit between August 2020 to and February 2021, according to the inclusion criteria. We excluded two visitors, who came for check-ups with no abnormal findings.

During their visits, all patients filled an SES form and reported the details of their systemic health using the Medical History/Health Questionnaire Form from the Center for Oral Medicine, College of Dental Medicine, Augusta University, Georgia, USA. Items in the SES included the highest education level achieved and the previous month's household income. Systemic health variables included a history of smoking, alcohol use, and chronic disease. These variables were used as exposures to analyze whether they were associated with OML outcomes.

The diagnostic criteria for OMLs were based on the fourth edition of *Oral and Maxillofacial Pathology* authored by Neville B, et al. In the dental office, an oral examination was performed under a dental chair light with the patient sitting on the chair. The specialist inspected and palpated using tools including a mirror, explorer, cotton swab, and sterile gauze. The specialist adopted the following examination sequence: face, lip, buccal, tongue, mouth floor, hard palate, soft palate, gum, and alveolar ridge. When the diagnosis was uncertain, we marked “diagnostic uncertainty” in the medical records due to a lack of oral pathology or other laboratory services in the rural hospital.

### Statistical Analysis

Age is presented as a median with interquartile range (IQR). Categorical variables are presented as numbers with percentages (%). Unadjusted or adjusted regression analyses were performed to analyze the risk factors (sex, age, education, the last-month household income, smoking, alcohol use, and chronic disease) associated with the presence of OMLs. These associations are reported as unadjusted or adjusted odds ratios (OR) with 95% confidence intervals (CI). Statistical analyses were conducted using SPSS software (version 26.0, IBM Corporation, Armonk, NY, USA). Statistical significance was set at *p* < 0.05.

## Results

A total of 146 patients were enrolled in this study, including 77 (52.7%) women and 69 (47.3%) men, with a median age of 51.5 years (IQR, 30.8–65.0 years). These patients were mostly Han ethnicity (*n* = 132, 90.4%), native residents (*n* = 134, 91.8%), and living in villages (*n* = 117, 80.1%). Approximately 0.03% (134/406,642^*^100%) native residents visited our clinic. Nearly 75% of the patients had not graduated from high school (*n* = 107, 77.0%) and did not earn more than 6,000 yuan as a household income in the previous month (*n* = 99, 73.9%). Approximately 25% of the patients had a history of smoking (*n* = 35, 24.8%) or alcohol use (*n* = 36, 25.5%).

Eighty-six (58.9%) patients had a history of chronic disease (Table 1). Table 2 shows the distribution of chronic diseases. The most common chronic diseases were sleep apnea (*n* = 35, 24.0%), hypertension (*n* = 23, 15.8%), bronchitis (including emphysema) (*n* = 16, 11.0%), reflux (including gastroesophageal reflux disease) (*n* = 12, 8.2%), and arthritis (*n* = 11, 7.5%). Forty-three (29.5%) patients had more than one chronic disease.

**Table 1:** Characteristics of patients.

**Variable**	***n***	**%**
Sex
Female	77	52.7
Male	69	47.3
Age, years, *N* (IQR)	51.5 (30.8–65.0)
Ethnicity
Han	132	90.4
Others	14	9.6
Education* (highest level achieved)
Less than high school	107	77.0
High school and above	32	23.0
Last-month household income*
<6,000 yuan	99	73.9
6,000 yuan and above	35	26.1
Primary residence
Native county	134	91.8
Nearby county or migrant	12	8.2
Settled place
Village	117	80.1
Town or city	29	19.9
Smoking*
No	106	75.2
Yes	35	24.8
Alcohol use*
No	105	74.5
Yes	36	25.5
Chronic disease
No	60	41.1
Yes	86	58.9

Data are shown as n (% of available data) or median (interquartile range). The total number of patients was 146. *Missing data: education, n = 7; last-month household income, n = 12; smoking, n = 5; alcohol use, n = 5.

**Table 2:** Distributions of chronic diseases.

**Chronic diseases**	***n***	**%**
Respiratory problems	55	37.7
Asthma	3	2.1
Tuberculosis	1	0.7
Sleep apnea	35	24.0
Bronchitis/emphysema	16	11.0
Hematologic problems	16	11.0
Anemia	8	5.5
HIV/AIDS	1	0.7
Warfarin treatment	3	2.1
Others	4	2.7
Cardiovascular problems	31	21.2
Hypertension	23	15.8
Angina/chest pain	1	0.7
Heart attack/myocardial infarction	1	0.7
Prosthetic (artificial) heart valve	1	0.7
Others	5	3.4
Gastrointestinal problems	20	13.7
Hepatitis/jaundice	2	1.4
GERD/reflux	12	8.2
Others	6	4.1
Neurological problems	19	13.0
Stroke/TIA/mini-stroke	9	6.2
Neuropathy/neuropathic pain	4	2.7
Others	6	4.1
Endocrine problems	14	9.6
Diabetes	9	6.2
Thyroid disorder	3	2.1
Others	2	1.4
Kidney disease or dialysis	2	1.4
Cancer	1	0.7
Radiation therapy	1	0.7
Chemotherapy	1	0.7
Arthritis	11	7.5
Sjögren's syndrome	1	0.7

*AIDS, acquired immunodeficiency syndrome; GERD, gastroesophageal reflux disease; HIV, human immunodeficiency virus; TIA, transient ischemic attack*.

A total of 23 types of OMLs were found in 146 patients, including three patients who had two types of OMLs. The most common OMLs were aphthous ulcer (*n* = 41, 28.1%), burning mouth syndrome (BMS) (*n* = 16, 11.0%), viral ulcer (*n* = 14, 9.6%), traumatic ulcer (*n* = 13, 8.9%), and oral lichen planus (OLP) (*n* = 9, 6.2%) (Table 3).

**Table 3:** Frequencies of oral mucosal lesions.

**Oral mucosal lesions**	***n***	**%**
Allergy	3	2.1
Angular cheilitis	2	1.4
Bacteria	1	0.7
Burning mouth syndrome	16	11.0
Fungus	4	2.7
Geographic tongue	3	2.1
Gingival condition	2	1.4
Glossitis	4	2.7
Hematoma	4	2.7
Hyperkeratosis	1	0.7
Lump	6	4.1
Mucositis	2	1.4
Neuropathic pain	3	2.1
Oral cancer	2	1.4
Oral dryness	2	1.4
Oral lichen planus	9	6.2
Oral submucous fibrosis	1	0.7
Oral ulcer	80	54.8
Aphthous ulcer	41	28.1
Traumatic ulcer	13	8.9
Viral ulcer	14	9.6
Unclassified ulcer	12	8.2
Pigmentation	1	0.7
Uncertainty	3	2.1

On unadjusted regression, the patients without chronic diseases had a 20% (95% CI, 0.04–0.8) lower risk of BMS than the patients with chronic diseases. After adjustment for sex, age, education, last-month household income, smoking, and alcohol use, those without chronic diseases still had a 20% (95% CI, 0.03–0.9) lower risk of BMS than those with chronic diseases. A similarly decreased risk (OR, 0.2; 95% CI, 0.05–0.9) of OLP was observed for the previous month's household income of <6,000 yuan adjusted for the covariates, including sex, age, education, smoking, alcohol use, and chronic diseases (Table 4). The protective factor independently associated with viral ulcer was an older age (OR, 1.0; 95% CI, 0.9–1.0). Non-smoking was associated with a significantly increased risk (OR, 2.9; 95% CI, 1.0–8.0) of aphthous ulcer on unadjusted regression. After adjustment for sex, age, education, last-month household income, alcohol use, and chronic disease, no association was found between smokers and non-smokers. Similarly, patients without chronic diseases were associated with an increased risk (OR, 4.1; 95% CI, 1.2–13.8) of developing viral ulcers on unadjusted regression. However, after adjusting the analysis for sex, age, education, last-month household income, smoking, and alcohol use, this association disappeared (Table 5).

**Table 4:** Characteristics associated with burning mouth syndrome or oral lichen planus.

**Characteristics**	**Burning mouth syndrome**				**Oral lichen planus**			
	**Unadjusted OR (95% CI)**	***p***	**Adjusted OR (95% CI)**	***p***	**Unadjusted OR (95% CI)**	***p***	**Adjusted OR (95% CI)**	***p***
**Sex**
Female	0.9 (0.3–2.4)	0.77	2.2 (0.4–13.3)	0.39	0.4 (0.1–1.8)	0.24	0.4 (0.1–2.4)	0.31
Age*	1.0 (1.0–1.0)	0.15	1.0 (1.0–1.0)	0.80	1.0 (1.0–1.1)	0.19	1.0 (1.0–1.1)	0.31
**Education** ^**†**^
< High school	4.7 (0.6–37.0)	0.14	3.8 (0.4–35.0)	0.24	1.0 (0.2–5.0)	0.98	1.4 (0.2–9.6)	0.74
**Income** ^**‡**^
<6,000 yuan	5.3 (0.7–41.8)	0.11	4.7 (0.6–38.3)	0.15	0.2 (0.1–0.9)	0.03	0.2 (0.05–0.9)	0.03
**Smoking**
No	0.7 (0.2–2.1)	0.47	0.6 (0.1–4.6)	0.66	0.6 (0.1–2.6)	0.50	1.4 (0.2–11.6)	0.76
**Alcohol use**
No	0.5 (0.2–1.5)	0.21	0.6 (0.2–2.6)	0.51	0.6 (0.2–2.7)	0.54	0.9 (0.1–6.3)	0.92
**Chronic diseases**
No	0.2 (0.04–0.8)	0.03	0.2 (0.03–0.9)	0.04	0.4 (0.1–1.9)	0.25	0.6 (0.1–4.1)	0.61

*OR, odds ratio; CI, confidence interval*.

*Age (years).

*^†^Education (highest level achieved).*

*^‡^Income (last-month household). Adjusted for sex, age, education, income, smoking, alcohol use, and chronic disease. The references were male gender, ≥high school education, ≥6,000 yuan income, yes to smoking, yes to alcohol, and yes to chronic diseases*.

**Table 5:** Characteristics associated with aphthous ulcer or viral ulcer.

	**Aphthous ulcer**				**Viral ulcer**			
	**Unadjusted OR (95% CI)**	***p***	**Adjusted OR (95% CI)**	***p***	**Unadjusted OR (95% CI)**	***p***	**Adjusted OR (95% CI)**	***p***
**Sex**
Female	2.1 (1.0–4.5)	0.05	1.7 (0.7–4.2)	0.29	1.7 (0.5–5.3)	0.37	3.6 (0.7–19.5)	0.14
Age*	1.0 (1.0–1.0)	0.68	1.0 (1.0–1.0)	0.70	1.0 (0.9–1.0)	0.01	1.0 (0.9–1.0)	0.04
**Education** ^**†**^
< High school	0.6 (0.2–1.3)	0.18	0.5 (0.2–1.3)	0.14	0.7 (0.2–2.3)	0.53	1.0 (0.2–3.6)	0.94
**Income** ^**‡**^
<6,000 yuan	1.0 (0.4–2.3)	0.94	1.0 (0.4–2.5)	0.96	0.8 (0.2–2.6)	0.67	1.0 (0.2–4.0)	0.99
**Smoking**
No	2.9 (1.0–8.0)	0.04	2.0 (0.5–8.0)	0.33	1.2 (0.3–4.5)	0.82	0.4 (0.03–3.9)	0.41
**Alcohol use**
No	1.9 (0.7–4.6)	0.19	1.0 (0.3–3.1)	0.99	0.8 (0.2–2.7)	0.72	0.5 (0.1–2.6)	0.40
**Chronic diseases**
No	1.3 (0.7–2.8)	0.42	1.3 (0.5–3.0)	0.61	4.1 (1.2–13.8)	0.02	2.6 (0.6–10.9)	0.18

*OR, odds ratio; CI, confidence interval*.

*Age (years).

*^†^Education (highest level achieved).*

*^‡^Income (last-month household). Adjusted for sex, age, education, income, smoking, alcohol use, and chronic disease. The references were male gender, ≥high school education, ≥6,000 yuan income, yes to smoking, yes to alcohol, and yes to chronic diseases*.

## Discussion

In this study, we found the most common OMLs were aphthous ulcer, BMS, and viral ulcer. The most common comorbidities were sleep apnea, hypertension, and bronchitis (including emphysema). Patients with chronic diseases had a higher prevalence of BMS compared without those without chronic diseases. A household income of <6,000 yuan was an independent risk factor for OLP. These major findings can provide practical information for the government to optimize medical resources during the prevention and intervention of OMLs in remote rural areas where oral services are limited.

According to our study objective and available resource, we only assess the frequency of the OMLs at the department of stomatology instead of the prevalence. In terms of the relative frequency of OMLs, findings vary in the literature due to different diagnostic criteria, participants, and study methods. Patients with or without pathogen cultures and histopathological examinations might have different diagnoses. The use of a community-based survey or a medical records review could also cause certain analysis biases, such as participants enrolled. Overall, aphthous ulcer, BMS, and OLP were the most frequent OMLs (9, 10). Oral submucous fibrosis was not found in our study, but its frequency is high in some regions of China, such as Hunan (11), probably due to the common habit of chewing betel nuts. For example, a single center study from Hunan in China shows the most common OMLs are as follows: aphthous ulcer (27.17%), BMS (15.72%), OSF (14.75%), and OLP (10.38%). The most frequent two of OMLs in Changsha City, Hunan is similar to our study. OSF is the third in the most common OMLs, whereas the counterpart is viral ulcer and traumatic ulcer in our study (11). The present study also showed that 24.0% our study population had sleep apnea, which was the most common comorbidity. This is consistent with the prevalence of sleep apnea in the general population (12). Other common comorbidities were hypertension, bronchitis (including emphysema), reflux (including gastroesophageal reflux disease), and arthritis. The life and work routines might explain the frequency of comorbid diseases. People in our survey area had a high prevalence of sleep apnea and were more likely to have frequent smoking and/or drinking habits, which increased their risk for hypertension. A certain number of people in our survey area smoked and worked in the mining industry; thus, it is common for them to have lung diseases. Reflux mostly affected people who work in mountains for extended periods and skipped their meals. Prolonged standing or lengthy climbing up and down during work increases the risk of arthritis. This finding about comorbidities was also consistent to the spectrum of chronic disease in a study from China (13).

Aphthous ulcer is among the most common OMLs, with a prevalence of ~20% (14) and an incidence ranging from 5 to 50% (15). Smoking is its protective factor (16, 17). Our study also supported such relationship between smoking and aphthous ulcer on unadjusted regression analysis. However, this association disappeared after adjusting for multiple confounders. The reason for this loss might be that the causes of aphthous ulcer were comprehensive and multifactorial, including a genetic background, stress, and nutritional deficiencies (18). Viral ulcers in the mouth affected more than 85% of adults (19). Additionally, 40% of patients with primary herpetic simplex virus (HSV) experienced recurrent HSV infection (20). There was a small significant age-related decrease in the frequency of viral ulcer after adjusting for sex, the highest education level achieved, the previous month's household income, smoking, alcohol use, and chronic diseases in our study. The reason for this might be because primary HSV infection was common in children aged 6 months to 5 years and young adults aged 20 years (21). Some associations were found between chronic disease and viral ulcer on unadjusted regression. The explanation for this association could be that recurrent HSV infection happened from its latent forms in the trigeminal ganglions during a host's immunocompromised or immunosuppressed state (22, 23). However, no interaction was identified between chronic disease and viral ulcer in the context of sex, age, highest education level achieved, previous-month household income, smoking, or alcohol use. It is notable that oral viral ulcers can also happen in patients without chronic diseases. The causal relationship between oral viral ulcers and chronic disease requires further investigation.

The prevalence of BMS in the general population was estimated to be between 0.7 and 8% based on different diagnostic criteria (24, 25). In the current study, we found that chronic disease increased the risk of developing BMS after adjusting for sex, age, highest education level achieved, previous-month household income, smoking, and alcohol use. The reason for this could be that systemic factors, including anemia, diabetes, thyroid disease, hormonal deficiency, upper respiratory tract infection, gastroesophageal reflex disease, Parkinson's disease, and side effects of antihypertensive medications, were associated with BMS (26).

OLP is a chronic inflammatory condition, which is a common mucocutaneous disorder in the oral cavity. The prevalence of OLP was estimated to be between 1 and 3% (27). Systemic factors, such as hypertension, diabetes, viral infection, autoimmunity or immunodeficiency, and cancer, were supposed to be its etiology (27). Patients with OLP had significantly higher prevalence of stress, anxiety, and depression than the general population (28–30). A chronic and long-lasting course of OLP could make patients stressed, anxious, and depressed. Meanwhile, stress, anxiety, and depression could lead to the development of OLP. Furthermore, there might be an association between depression and high income, although this suggestion contradicted other study results (31–33). People with high incomes may perform high-pressure work in a competitive environment. Depressed individuals with high income levels may have an increased risk for developing OLP. However, this association should be treated with caution because chronic disease can reduce the associations of SES factors, such as income, with depression (31–33). The underlying mechanism needs to be explored further.

Our study had some limitations. The lack of a representative sample is a major limitation. To reduce the bias, we included all consecutive participants in our study. The small sample size could cause biases in our result analysis. Clinically, some vesicular diseases of the oral cavity may share a similar appearance. We separated viral ulcer from other non-viral vesicular diseases, for example, aphthous ulcer, by performing clinical examination carefully and taking a health history thoroughly. Through obtaining a complete health history, including dental filling (lichenoid contact reactions), medicine use (drug-induced lichenoid), and transplantation (oral graft-vs.-host disease), we also differentiated OLP from other oral lichenoid lesions (OLLs). Although we took measures to arrive at an accurate diagnosis, some patients might have vesicular diseases or OLLs that were difficult to distinguish from viral ulcer or OLP without laboratory confirmation. Furthermore, we defined BMS according to a burning sensation of the oral mucosa without abnormal oral mucosal findings, lasting at least 3 months. By this, we excluded some transient burning sensation. However, some system diseases might complicate the precise diagnosis. Therefore, the frequency of viral ulcer, OLP, or BMS might be overestimated. All diagnoses were made by one single specialist. Thus, the reliability of the diagnoses might be weakened.

In conclusion, we demonstrated the frequency of different OMLs in rural Yunnan and found that aphthous ulcer was the most common OML, which was followed by BMS, viral ulcers, and OLP. Chronic diseases were associated with a significant increase in BMS, and a high-income level was associated with OLP. A personized intervention policy for OMLs might be an appropriate approach, due to the low rate of oral medicine clinic visits in Xiangyun County and the distinct epidemiological characteristics of the rural milieu. Priority of oral care policies should be given to patients with aphthous ulcer. Preventive interventions of BMS should be developed among people with chronic disease.

## Data Availability Statement

The raw data supporting the conclusions of this article will be made available by the authors, without undue reservation.

## Ethics Statement

The studies involving human participants were reviewed and approved by the Ethics Committee of the People's Hospital of Xiangyun (no. 2020069). Written informed consent to participate in this study was provided by the participants and/or their legal guardian/next of kin.

## Author Contributions

HY, ML, and GT formulated the conception and designed the study. HY, QZ, QS, and ML acquired, analyzed, and interpreted the data. GT and HY drafted the manuscript. All authors have reviewed and approved the manuscript.

## Funding

This study was supported by Research Funding of the People's Hospital of Xiangyun (Grant No. DX2020SF02).

## Conflict of Interest

The authors declare that the research was conducted in the absence of any commercial or financial relationships that could be construed as a potential conflict of interest.

## Publisher's Note

All claims expressed in this article are solely those of the authors and do not necessarily represent those of their affiliated organizations, or those of the publisher, the editors and the reviewers. Any product that may be evaluated in this article, or claim that may be made by its manufacturer, is not guaranteed or endorsed by the publisher.
